# Imaging Findings of Vigabatrin-Associated Neurotoxicity in a 12-Month-Old With Infantile Epileptic Spasm Syndrome

**DOI:** 10.7759/cureus.88593

**Published:** 2025-07-23

**Authors:** Jennifer Nedimyer Horner, Mohamad A Asfour, Gleidson Silva, Tushar Chandra

**Affiliations:** 1 Radiology, University of Central Florida College of Medicine, Orlando, USA; 2 Diagnostic Radiology, Hospital Corporation of America (HCA) Healthcare/University of South Florida (USF) Morsani College of Medicine, Trinity, USA; 3 Pediatric Neuroradiology, Nemours Children's Hospital, Orlando, USA

**Keywords:** anterior commissure, globi pallidi, infantile epileptic spasm syndrome, medial thalami, mri, neuroradiology, neurotoxicity, restricted diffusion, vigabatrin, west syndrome

## Abstract

Infantile epileptic spasm syndrome (IESS), also known as West syndrome, is a rare and severe form of epileptic encephalopathy of infancy. Vigabatrin, a selective, irreversible inhibitor of gamma-aminobutyric acid transaminase (GABA-T), is currently the only FDA-approved medication for the management of IESS. Known associations include neurotoxicity and visual defects.

A 12-month-old female with a history of developmental delay and IESS, currently being treated with vigabatrin, was evaluated with magnetic resonance imaging (MRI) after presenting with excessive drowsiness and poor feeding. Imaging findings were significant for new areas of restricted diffusion involving the anterior commissure, medial aspect of the globi pallidi, and the medial thalami bilaterally compared to the patient’s MRI prior to starting vigabatrin. These findings suggested vigabatrin neurotoxicity.

This case demonstrates the clear relationship between vigabatrin use and the development of stereotypical imaging abnormalities associated with neurotoxicity. It also further illustrates the importance of early recognition and withdrawal of therapy in order to prevent potential long-term side effects.

## Introduction

Seizure disorders affect nearly three million Americans of all ages, with roughly 150,000 new cases diagnosed each year [1]. Seizures are caused by abnormally increased or synchronized electrical conduction in the brain. There are multiple types of seizures, and they are classified based on the region of brain involvement as well as the movements observed during the seizure [1]. Seizures can be provoked or unprovoked, and the term “epilepsy” is usually used to describe cases in which a patient has two or more unprovoked seizures or one unprovoked seizure with a high likelihood for recurrence based on either abnormal diagnostic imaging findings, genetics, or a positive family history for seizure disorders [1]. 

Although still an evolving field of study, one important underlying cause attributed to seizures is decreased levels of gamma-aminobutyric acid (GABA) in the central nervous system [2]. GABA is an important inhibitory neurotransmitter involved in reducing neuronal excitability. It is metabolized in the body by an enzyme called GABA transaminase (GABA-T), and so one method of pharmaceutical intervention is to inhibit the GABA-T enzyme, thereby increasing the GABA levels in the central nervous system.

Infantile epileptic spasm syndrome (IESS), also known as West syndrome, is a seizure disorder affecting infants (M>F) between the ages of one and 12 months [1]. It is a rare disorder with an incidence rate of 1.6 to 4.5 cases per 10,000 live births [3]. In the United States, approximately 2000 to 2500 new cases are diagnosed annually, with an average onset between three and seven months of age [3]. Since it was first described in 1841 by William James West, ongoing research on the syndrome’s pathophysiology has uncovered a broad spectrum of causal factors, including infectious, structural, immunologic, and metabolic insults occurring at various points during the prenatal, perinatal, or postnatal period [4]. These insults have been observed independently or in combination to act on a background of genetic predisposition, culminating in the characteristic triad of IESS: infantile spasms, hypsarrhythmia, and developmental regression or arrest [4]. The condition has been linked to abnormalities in the hypothalamic-pituitary-adrenal axis based on studies that have uncovered responsiveness to treatment with adrenocorticotropic hormone (ACTH), which remains the first-line treatment. It has also been associated with multiple genetic disorders, including tuberous sclerosis, Trisomy 21, CDKL5 deficiency disorder, duplication 15q syndrome, and others [4].

In 2009, vigabatrin became the first and currently only traditional anti-epileptic medication approved for the treatment of IESS. As a GABA-T inhibitor, it prevents the degradation of GABA in the brain, thereby increasing levels of GABA in the brain and reducing neuronal excitability [5]. It is used as a second line to hormonal therapy and has been shown to be especially effective in treating IESS in association with tuberous sclerosis. Although the most common side effects associated with vigabatrin are sedation and fatigue, it has also been linked to vision loss and neurotoxicity [5]. As a result, patients require routinely scheduled eye exams and brain MRIs to monitor for side effects related to drug toxicity.

This case study describes a 12-month-old female with early developmental delay who was diagnosed with hormone-refractory IESS. She received treatment with vigabatrin and was subsequently found to have neuroradiological findings on MRI consistent with the distinctive vigabatrin neurotoxicity pattern that has been described in the literature.

## Case presentation

The patient was born via an uncomplicated scheduled cesarean section at 38 weeks of gestation. She first presented to the clinic at five months for concerns of developmental delay due to inadequate growth for her age and poor visual tracking observed by the patient’s family. Her family denied any recent illnesses and reported that she was up-to-date on all age-appropriate vaccines. Physical exam findings were normal apart from plagiocephaly and torticollis with a left-sided preference. An MRI of the brain was obtained at this time and was normal. The patient was referred for evaluation by an ophthalmologist who noted inconsistent visual fixation. The family was educated about delayed visual maturation and asked to follow up in three months.

At eight months of age, the patient presented to the emergency department for evaluation of new seizure-like activity. The family described several episodes at home of stiffening of her extremities, back arching, and eyes rolling back. These episodes were witnessed in the emergency department, resulting in admission and video EEG, which confirmed seizure activity consistent with IESS. The patient was treated appropriately in the inpatient setting and discharged three days later on phenobarbital, high-dose corticosteroids with a 14-day taper, enalapril for hypertension, and vitamin B6 supplementation. She complied with regular outpatient follow-up for close blood pressure monitoring and completed her corticosteroid course.

At 10 months, the patient was offered to start ACTH, which is first-line therapy for IESS, or vigabatrin. The risks and benefits of each were discussed. In consideration of the patient’s significant weight gain from past steroids and her persisting hypertension, the family elected to start vigabatrin. Vigabatrin was started and titrated to 150 mg/kg/day, and lacosamide was subsequently weaned after five days. Keppra was continued.

**Figure 1:**
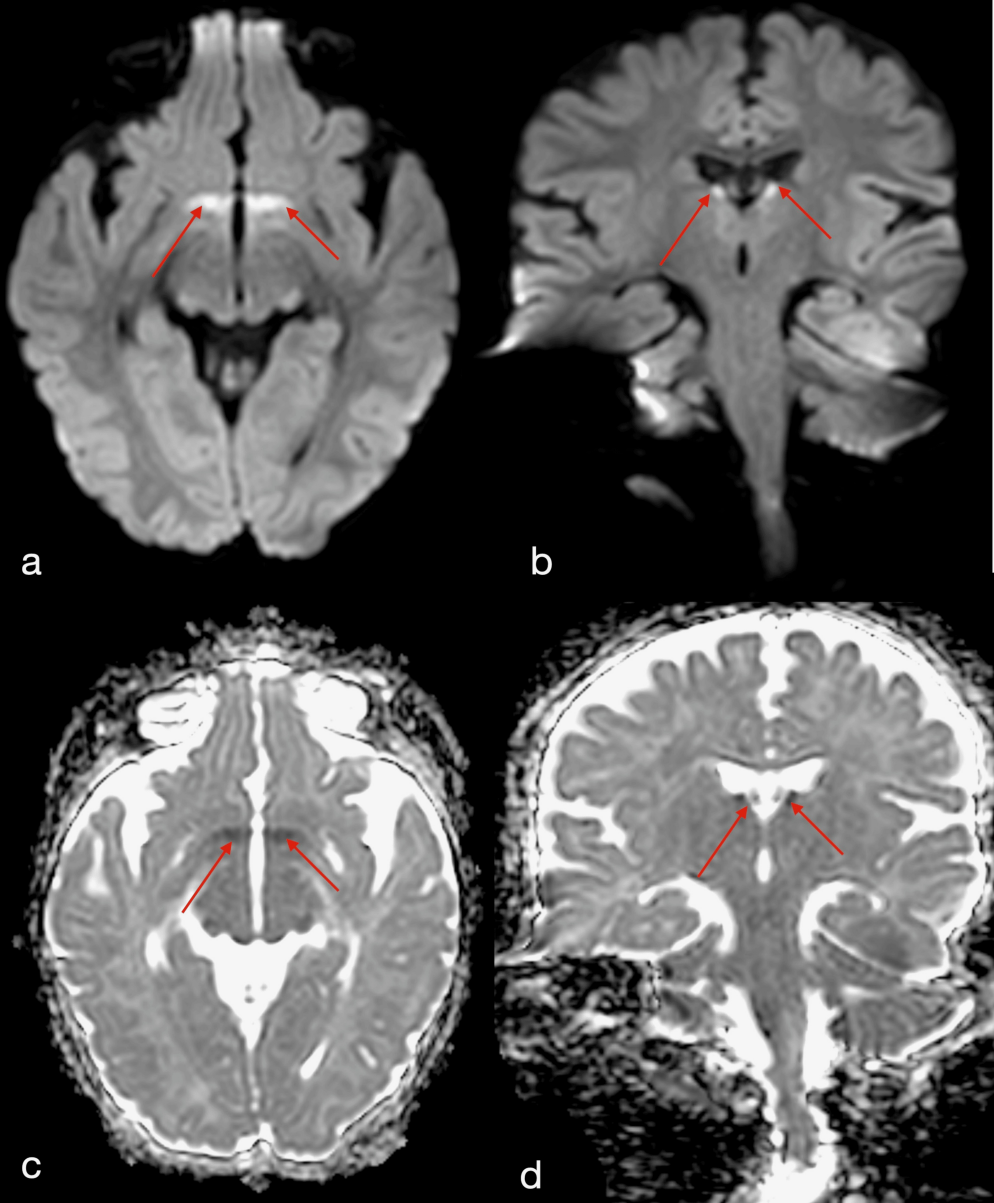
Axial and coronal DWI and ADC brain MRI of the anterior commissure Axial and coronal images demonstrate hyperintense signal on DWI (a and b) and loss of signal on ADC (c and d) in the bilateral anterior commissure (red arrows) to suggest restricted diffusion. DWI: diffusion-weighted imaging; ADC: apparent diffusion coefficient

**Figure 2:**
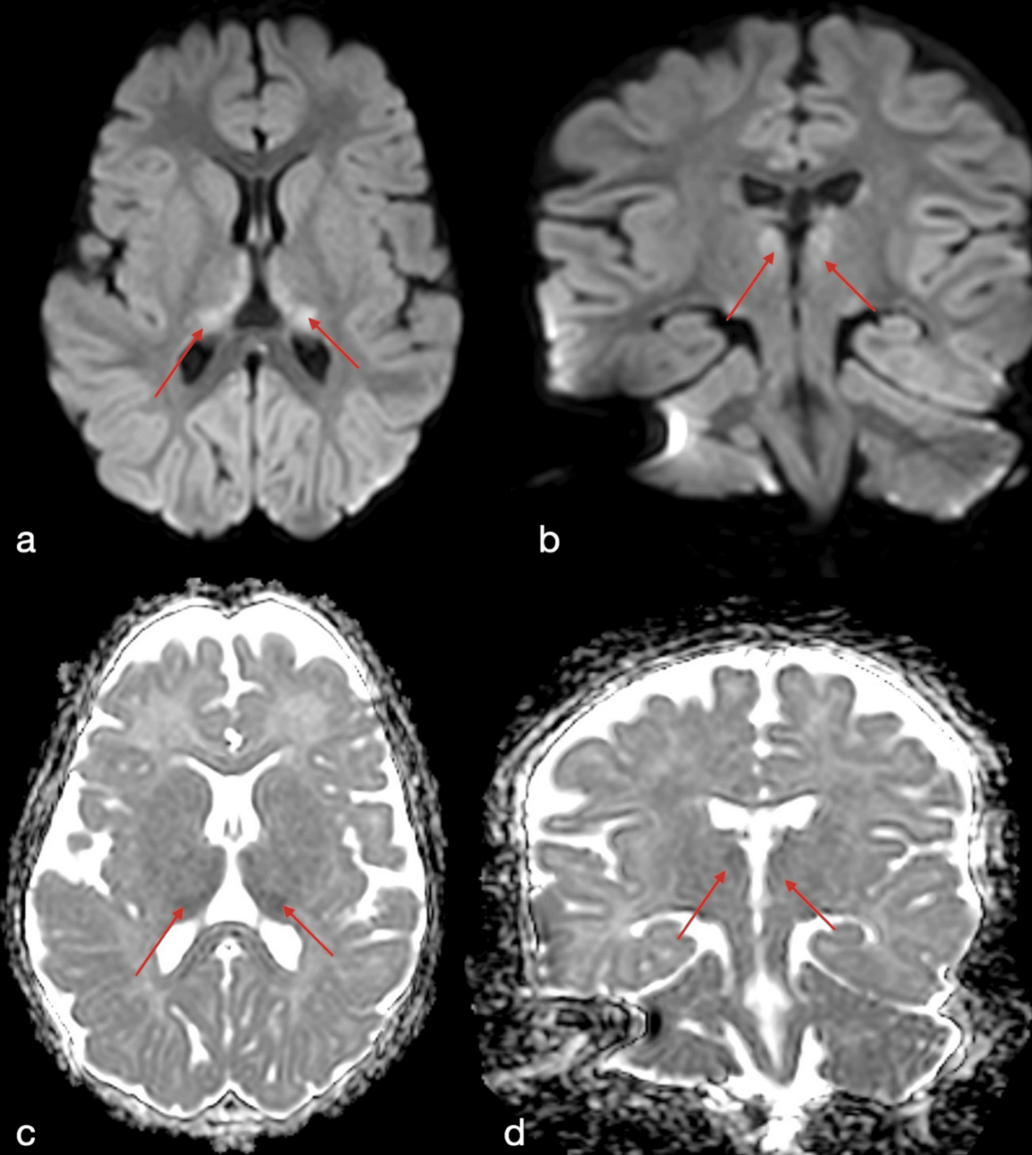
Axial and coronal DWI and ADC brain MRI of the thalami Axial and coronal images demonstrate hyperintense signal on DWI (a and b) and loss of signal on ADC (c and d) in the bilateral paramedian thalami (red arrows) to suggest restricted diffusion. DWI: diffusion-weighted imaging; ADC: apparent diffusion coefficient

The patient was seen at follow-up two months later at 12 months of age. Her family noted that the spasms continued but at a reduced frequency. They reported no focal or tonic seizures over the past two months but expressed concern for the patient’s excessive sleepiness and poor feeding. An MRI was ordered, and the results demonstrated areas of restricted diffusion involving the anterior commissure (Figure 1), the medial aspect of the globi pallidi, and the medial thalami bilaterally (Figure 2). These findings are consistent with vigabatrin-associated neurotoxicity as described in the literature. The results and treatment options were discussed with the patient’s family, and the decision was made to discontinue vigabatrin treatment. One month later, the patient's family reported improvement in symptoms. A repeat MRI has been deferred at this time, as the risks of sedation MRI were deemed to outweigh the benefits. 

## Discussion

Treatment-related neurotoxicity is a term that describes a group of clinical conditions in which the causative agent is a medication used in the treatment of a particular disease. Recognition of certain patterns is crucial, especially in the neonatal period, where neurological symptoms may be nonspecific yet have detrimental results [6]. The known mechanisms of action of therapeutic agents and treatment methods, when used alone or with a few other medications, aid in the identification of MRI patterns, such as in the deep gray matter nuclei or white matter.

Vigabatrin is a selective and irreversible inhibitor of γ-aminobutyric acid transaminase (GABA-T) and is a commonly used anti-epileptic drug in infants [7]. This is because vigabatrin is known for its good tolerability and overall lack of drug-on-drug interactions, except for notably phenytoin. Retinal toxicity with visual field defects, however, is seen in up to 30% of patients after one year of use. Asymptomatic MRI abnormalities while on therapy are seen in 10-20% of infants as well and are thought to be related to a GABA-induced excitotoxicity mechanism [6]. The appearance of restricted diffusion should be taken seriously, especially when accompanied by new or worsening neurological symptoms.

Although the exact mechanism of underlying vigabatrin-induced neurotoxicity remains unclear, a widely supported theory suggests that excess accumulation of GABA in the immature brain interferes with the tightly regulated process of synaptogenesis and neuronal differentiation, predominately in areas of high GABA receptor density such as the thalami, basal ganglia, and brainstem [8]. Additionally, these studies suggest that increased GABA may potentially lead to cytotoxic edema and thus restricted diffusion as seen on MRI [8].

The stereotypical MRI vigabatrin neurotoxicity pattern, like other drug-induced toxicities such as metronidazole, is characterized by basal ganglia, corpus callosum, dentate nuclei, and brainstem involvement. More rarely, the cerebellum and thalamus can be involved. In our case, this is suggested by the appearance of restricted diffusion at the bilateral paramedian thalami (Figure 2) and anterior commissure (Figure 1) without corresponding T1 or T2 signal abnormalities, similar to previously reported findings [7]. Previous studies have also shown that these changes may occur without clinical symptoms [7].

A 2010 study by Pearl et al. found that in 16% of infants on vigabatrin, MRI abnormalities appeared in deep nuclei, often with reversible findings upon cessation of the drug [8]. This supports our decision to halt therapy, which has gradually led to symptom improvement. Additional studies have also demonstrated that the resolution of MRI findings often correlates with clinical recovery [9,10]. There have, however, been some cases of persistent developmental delay even after cessation of drug therapy, thus suggesting potential long-term subclinical effects [9,10]. 

The differential diagnosis for these lesions is broad and includes conditions such as metabolic disorders, mitochondrial diseases, and Leigh syndrome, just to name a few. A detailed medical and medication history is essential to help narrow the diagnosis, avoid unnecessary metabolic and genetic testing, prevent delays in appropriate care, and reduce the financial burden on the family. 

The pathophysiology of these MRI abnormalities remains incompletely understood. Symmetrical involvement in these deep gray matter structures in patients treated with vigabatrin is a strong indicator of vigabatrin toxicity. Importantly, these findings are often reversible with discontinuation of therapy. More longitudinal studies are needed to evaluate the cognitive and developmental outcomes of patients who experience vigabatrin-related neurotoxicity. 

## Conclusions

Vigabatrin-associated neurotoxicity should be considered in the differential diagnosis for neonates with a history of vigabatrin treatment and MRI findings that demonstrate areas of restricted diffusion involving the anterior commissure, medial aspect of the globi pallidi, and the medial thalami bilaterally. While similar radiological findings may be associated with metabolic, infectious, or drug-induced leukoencephalopathies, the reading radiologist should seek to rule out vigabatrin-associated neurotoxicity early on. This might prevent costly and unnecessary diagnostic testing for the patient, which can make a significant difference in the outcome for the pediatric patient and the family. Furthermore, early recognition of this condition allows for the timely discontinuation of vigabatrin and the subsequent exploration of alternative treatment options.
